# Assessment of Knowledge and Attitude of General Practitioners Regarding Autism and Associated Factors at Gondar University Hospital, Gondar, Ethiopia

**DOI:** 10.1155/2024/9917927

**Published:** 2024-10-28

**Authors:** Assefa Kebad Mengesha, Alemante Tafese Beyna, Gemtew Enyew Kidanu, Melshew Fenta Misker, Habtamu Semagne Ayele

**Affiliations:** Department of Pharmacology, School of Pharmacy, University of Gondar, Gondar, Ethiopia

**Keywords:** attitude, autism spectrum disorder, Ethiopia, general practitioners, Gondar, knowledge

## Abstract

**Background:** The neurodevelopmental conditions known as autism spectrum disorders (ASDs) start in early childhood and last the entirety of a person's life. They are characterized by distorted social interaction, difficulties communicating, and repetitive, stereotypical behavior.

**Objective:** This study sought to evaluate general practitioners (GPs)' attitudes and knowledge of ASDs and related factors at Gondar University Hospital.

**Methods:** An institutional-based cross-sectional study design was used. Using a convenience-sample method, three-hundred sixty individuals were chosen for this study. Data were collected using a self-administered questionnaire. The GPs who took part in this survey were characterized by descriptive statistics. The relationship between the dependent variables (knowledge and attitude) and the sociodemographic characteristics was examined using an independent two-sample *t*-test and Pearson correlation analysis. The Statistical Package for the Social Sciences Version 25 was used for all data analyses.

**Results:** In this study, the GPs had an average age of 31.82 years, with an average of 18 years since graduation and 16 years of practice. Participants' average overall knowledge and attitude scores on autism were 15.83 (SD = 3.27) and 29.54 (SD = 3.21), respectively, both falling within the moderate range. Using an independent *t*-test, we found a significant difference (*p* < 0.001) between the attitudes of male and female GPs regarding autism. The study also identified weakly significant correlations between GPs' age and their attitudes toward autism (*r* = 0.271⁣^∗∗^, *p* < 0.001) and between years of practice and attitudes (*r* = 0.105⁣^∗^, *p*=0.046). However, no significant correlations emerged between GPs' knowledge and their age, years since graduation, or years of practice (*r* = 0.069, *p*=0.194; *r* = 0.069, *p*=0.193; and *r* = −0.053, *p*=0.312, respectively). In addition, we observed a weakly significant association (*r* = 0.004⁣^∗∗^, *p* < 0.001) between GPs' knowledge and their attitudes about autism.

**Conclusion:** Based on their total scores, the participants had a moderate level of knowledge and attitudes toward autism. There was a favorable correlation found between the study subjects' attitudes regarding autism and their age, as well as their practice year. Furthermore, a clear correlation was observed between GPs' attitudes and their understanding of autism.

## 1. Introduction

Neurodevelopmental diseases known as autism spectrum disorders (ASDs) are characterized by abnormal social interaction, communication difficulties, and repetitive, stereotypical behavior that start in early childhood and last the rest of a person's life [1–3]. The three types of etiology for autism are idiopathic (ASD without evidence of other neurological or biological problems), cryptogenic (an underlying cause is hypothesized), and symptomatic (unrecognized organic or neurological cause) [4–7].

Prenatal, natal, or postnatal problems are linked to genetic, environmental, or physical risk factors that can result in the development of ASDs [8–11]. Prenatal concerns include maternal factors such as advanced age, a preconception diet lacking in vitamins or folic acid, infection during pregnancy, and chronic and metabolic illnesses [12–14]. Postnatal risks include living in polluted or high-altitude settings, while natal risks include crying out too soon or too late [15–17]. The chance of developing autism in children is strongly correlated with the advanced age of the father, as evidenced by recent studies [18–20].

Males are five times more likely than females to have ASD; however, the frequency varies with sex [21, 22]. In addition, the frequency of the condition varies by race and area of residence [23, 24]. Several investigations confirm that ASD frequently affects individuals with white skin tones, metropolitan dwellers, and residents of areas with high air pollution [25–27].

SD affects over 7.6 million individuals with disabilities worldwide, with a prevalence of one in 160 people [28]. The data and patterns indicate that the rate of increase in autism prevalence is concerning [27, 29]. In comparison with numbers from a decade ago, the estimated prevalence of autism grew by an astounding 78%, according to a 2012 Centers for Disease Control and Prevention (CDC) report [30]. Two U.S. studies, conducted between 2009 and 2011 and between 2015 and 2017, observed a significant increase in the severity of developmental disabilities, including ASDs. In addition, there are notable differences in prevalence globally, with Middle Eastern countries reporting between 1.4 and 29 cases per 10,000 children [31].

Various research results indicate that industrialized countries are more likely than developing nations to experience this disease [27, 32–34]. However, there is insufficient data to draw any conclusions for the latter group [18]. Clinicians currently use the Diagnostic and Statistical Manual of Mental Disorders, Fifth Edition (DSM-5), to diagnose autism [35]. Two domains are necessary for the diagnosis of autism, according to this manual. Domain one includes social communication and social engagement, while domain two focuses on restricted and repetitive behavior patterns. The diagnostic criteria for autism consist of two criteria from domain two and three criteria from domain one. However, because autism symptoms can resemble those of other conditions, such as attention-deficit-hyperactivity disorder, clinicians may initially misdiagnose children with autism as having those conditions [36–39].

Health practitioners in several poor nations often fail to diagnose autism because they are unaware of the condition's genesis and diagnostic standards [40–43]. A pilot study from the previous research found that most general practitioners (GPs) lack expertise in the behavioral and developmental issues affecting children [44]. To support effective autism monitoring and research initiatives, improving health professionals' understanding of autism in developing nations is crucial.

In Ethiopia, there is limited research on autism, and healthcare professionals often lack adequate training and resources to diagnose and manage ASDs effectively. This study aims to evaluate the knowledge and attitudes of GPs at Gondar University Hospital regarding ASDs and the factors associated with them. Understanding the current state of knowledge and attitudes among GPs can inform the development of targeted training programs and policies to enhance autism care in Ethiopia. This research is necessary to address the gaps in autism awareness and diagnostic capabilities in the local healthcare context, ultimately improving the quality of care for individuals with ASDs in Ethiopia.

## 2. Methods

### 2.1. Study Area and Period

The research team conducted the study at Gondar University Hospital, which is located in the northwest Amhara Region of Ethiopia, 734 kilometers from Addis Ababa, the country's capital. The research team conducted the study over a two-month period, from May 1, 2023, to June 30, 2023.

### 2.2. Study Design

The researchers used an institutional-based cross-sectional study design.

### 2.3. Source Population

The study's source population comprised all GPs at Gondar University Hospital in Gondar, Ethiopia.

### 2.4. Study Population

GPs working at Gondar University Hospital, Gondar, Ethiopia, who volunteered to participate in the study.

### 2.5. Sampling Technique

**Figure 1:**
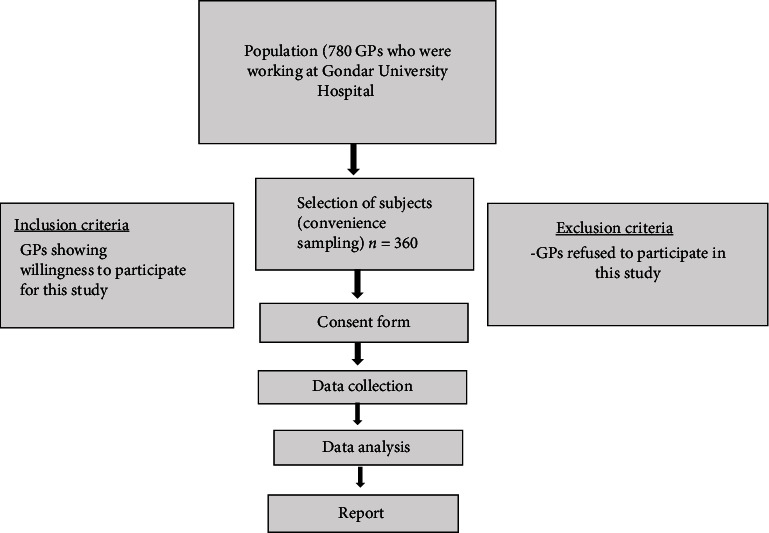
Flowchart of the research methodology.

This study used a convenience sampling strategy, selecting readily accessible subjects. At Gondar University Hospital, this approach allowed us to easily reach GPs who were available and willing to participate. This method was particularly practical, given the busy schedules of healthcare professionals. It is less time-consuming and more cost-effective than probability sampling, making it ideal for a study with limited time and resources. As an exploratory study, it aimed to provide preliminary insights into the knowledge and attitudes of GPs regarding ASDs, serving as a foundation for more comprehensive future research. Focusing on GPs at a central healthcare facility offered valuable insights that are applicable to similar settings across Ethiopia. Practical constraints, including difficulties in reaching a randomized sample, made convenience sampling a feasible solution, ensuring a higher response rate by targeting those more readily willing to participate. We selected 360 GPs employed at Gondar University Hospital based primarily on their availability (Figure 1).

Data collection instruments, procedures, and analysis. We gathered data using structured, self-administered questionnaires. Four questions in Part I addressed the socioeconomic traits of the individual GPs. We derived Parts II and III, which included ten and five items, respectively, from the Knowledge and Attitude about Childhood Autism among Health Workers (KCAHWs) questionnaire. A five-level Likert-item with the following values (strongly disagree, disagree, undecided, agree, and strongly agree) was part of the questionnaire for this study. We based the knowledge and attitude rating systems on earlier research [45–49]. The numbers were, in order, 1, 2, 3, 4, and 5. The specialists assessed the questionnaire. When drafting the final version of the questionnaire, we considered their input. We conducted all methods in accordance with relevant guidelines and regulations.

**Figure 2:**
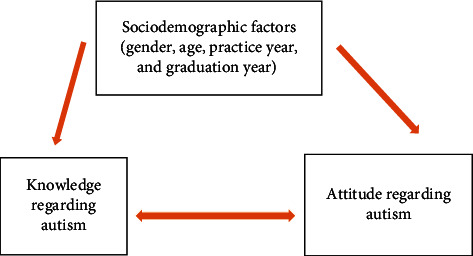
Conceptual framework on the relationship between knowledge and attitude regarding autism and the sociodemographic factors.

We characterized the GPs who participated in this survey using descriptive statistics. We examined the relationship between gender and autism knowledge scores using independent two-sample *t*-tests. We verified the relationship between age, years of practice, and graduation year with the dependent variables (knowledge and attitude) using a Pearson correlation test. We used the Statistical Package for the Social Sciences (SPSS) Version 25 for all data analyses (Figure 2).

## 3. Results

### 3.1. Demographic Profile of GPs Participating in a Study on Autism Knowledge and Attitudes

In this study, we surveyed 360 GPs. Of the participants, 58.9% were male (212 individuals) and 41.1% were female (148 individuals).

Regarding age groups, the majority of participants, constituting 51.1%, fell into the age group of under 31 years (184 individuals). A significant proportion, 33.3%, belonged to the 31–40 age group (120 individuals), and the remaining 15.6% were aged 40 years and older (56 individuals).

In terms of graduation years, 31.7% of the participants graduated less than 10 years ago (114 individuals). Those who graduated between 10 and 15 years ago comprised 14.7% (53 individuals), while a smaller percentage, 3.3% (12 individuals), graduated between 15 and 20 years ago. A substantial majority, 50.3% (181 individuals), had graduated 20 years ago or more.

Furthermore, 33.9% of the participants (122 individuals) had less than 10 years of experience. Those with 10–15 years of practice made up 13.3% (48 individuals), while only 4.7% (17 individuals) had 15–20 years of experience. The largest group, at 48.1% (173 individuals), had practiced for 20 years or more (Table 1).

### 3.2. Knowledge and Attitudes of the GPs Toward Autism

When asked whether the statement “Autistic children show detachment from their parents” was true, 33.6% of GPs agreed. In contrast, only 28.1% disagreed, 15.3% strongly disagreed, and 20.3% were unsure about the claim. In addition, when questioned about the misleading notion that autism is more common among higher socioeconomic strata, 20.3% of GPs were unsure. Similarly, when asked whether autism is more common among individuals with higher educational levels, only 31.4% of respondents disagreed, 16.7% strongly disagreed, and 13.3% were unsure of their position.  Table 2 provides additional summaries of the responses.

### 3.3. Evaluation of Knowledge and Attitude on Autism Among GPs at Gondar University Hospitals

Table 3 shows the total scores of knowledge and attitude regarding autism at different levels. The participants' mean overall knowledge and attitude scores on autism were 29.54 (SD = 3.205752) and 15.83 (SD = 3.265), respectively, falling in the moderate ranges.

### 3.4. Relationship Between Gender and GPs' Attitudes and Knowledge of Autism

Regarding knowledge scores, males had a mean of 29.29 with a standard deviation of 3.54, whereas females had a mean of 29.90 with a standard deviation of 2.63. The *t*-statistic for this comparison was −1.783 with 358 degrees of freedom, and the *p* value was 0.075. This result suggests that there is no statistically significant difference in knowledge scores between male and female practitioners, as the *p* value exceeds the commonly accepted threshold of 0.05.

In terms of attitude, the mean score for males was 16.34 (SD = 3.20), whereas females had a mean score of 15.08 (SD = 3.22). The *t*-statistic for this comparison was 3.675 with 358 degrees of freedom, and the *p* value was less than 0.001. This indicates a statistically significant difference, with males showing a more positive attitude toward autism compared to females.

### 3.5. Correlation Between Demographic Factors and GPs' Knowledge and Attitudes Regarding Autism

The Pearson correlation test showed no significant correlation between GPs' age, years since graduation, and years of practice with their knowledge about autism (*r* = 0.069, *p*=0.194; *r* = 0.069, *p*=0.193; and *r* = −0.053, *p*=0.312, respectively). However, the analysis revealed weakly significant relationships between age and views toward autism (*r* = 0.271⁣^∗∗^, *p* < 0.001) and between years of practice and views toward autism (*r* = 0.105⁣^∗^, *p*=0.046). Years of graduation did not show any meaningful association with perspectives on autism. In addition, we observed a small positive correlation (*r* = 0.004⁣^∗∗^, *p* < 0.001) between GPs' knowledge and their attitudes toward autism (Table 4).

## 4. Discussion

Recent epidemiological studies contradict the presented information by showing that autism is common across all socioeconomic and educational classes [50]. In addition, when asked if they agreed with the statement that “Autism is a precursor for schizophrenia,” only 19.2% disagreed, 16.7% strongly disagreed, and 31.1% were unsure. These opinions are in line with the descriptions of autistic children [51]. Recent studies have revealed that individuals with schizophrenia are substantially less likely to exhibit autistic disorder symptoms [52]. It is interesting to note that 33.6% and 25.6%, respectively, strongly agreed with the untrue assertion, “Children with autism often detach themselves from their family and peers.” This perspective is based on an antiquated idea that Kanner in 1943 proposed [53]. Contemporary research indicates that a combination of environmental and genetic factors causes autism to be a multifactorial illness.

Thus, frigid parental connection and childhood neglect cannot adequately account for autism [54, 55]. Finally, of those who responded to the incorrect assertion, “Autism is preventable,” only 28.6% disagreed and 21.7% strongly disagreed. In a related study, researchers asked 111 health professionals including 20 pediatricians, 8 family practitioners, 18 psychiatrists, 5 neurologists, 9 speech pathologists, 16 clinical psychologists, and 35 other medical doctors or professionals (PhD, MS) working for the Center for Autism and Related Disabilities about their knowledge and beliefs regarding autism. The results showed that the beliefs of family practitioners, pediatricians, and neurologists are consistent with out-of-date information about autism. For instance, there was a greater likelihood of agreement among family practitioners, pediatricians, and neurologists about the association between autism and higher socioeconomic and educational classes. Moreover, compared to specialists, psychiatrists, and speech pathologists, primary healthcare practitioners were considerably more likely to believe that parenting styles and parental psychopathology are the root causes of autism [56]. “It can be challenging to differentiate between autism and schizophrenia,” stated 30.0% of respondents strongly.

Previous prevalent research has revealed that a notable proportion of GPs in the region exhibit inadequacies in diagnosing or treating common medical conditions like tuberculosis or acute respiratory infections [57, 58]. Few studies examine the stigmatizing beliefs doctors hold about ASD, and those that exist do not explore the potential causes or influences of these attitudes. The Dutch physicians who participated in the study, however, had a more stigmatizing attitude toward ASD than Western physicians but a lower attitude than non-Western physicians, according to a study of 93 youth- and family-center doctors from the Netherlands [59]. Since the measurement instruments and analytical techniques used in this investigation differ greatly from ours, it is regrettably impossible to compare the two (Table 2).

For a number of reasons, including improved service delivery [60, 61] for children with ASD and early case identification for suitable therapies [62], a greater understanding of ASD among GPs is crucial. Furthermore, improved GPs' understanding of ASD can boost patient, parent, and physician trust [63]. As doctors gain more expertise, they will be better equipped to educate future generations of physicians. This study evaluated the level of knowledge and attitude toward autism among GPs (*n* = 360) at Gondar University Hospital. The participants' mean overall knowledge and attitude scores on autism fell into the moderate ranges. Similar to our survey, a study of 93 youth- and family-centered physicians from the Netherlands that assessed the physicians' knowledge about ASD and the stigmatizing attitude they held toward ASD and mental illness found that the physicians had sufficient general knowledge about ASD [64]. In addition, a study of 304 GPs from the United Kingdom found similar results in that GPs had adequate knowledge about ASD [60] (Table 3). Moderate knowledge and attitudes among GPs can delay autism diagnoses and lead to less effective treatment, negatively impacting patient care and family support.

Continuing Medical Education (CME) courses and interactive workshops: Developing comprehensive educational resources and conducting awareness campaigns will improve understanding. Encouraging interdisciplinary collaboration and establishing peer support networks can further support GPs. Regular knowledge assessments and feedback from patients and families will help identify gaps and areas for improvement. Integrating autism education into medical school curricula and residency programs will prepare future GPs better. Securing funding for the continuous development of educational materials and resources will ensure ongoing improvement and effectiveness.

The present investigation examined the impact of gender on the degree of knowledge and attitude regarding the characteristics and identification of ASD. The results of the independent *t*-test indicated that there was no significant correlation between the genders and the GPs' knowledge about autism. This finding matches the results of a study that researchers conducted in New South Wales (NSW), Australia [65]. However, a different study revealed a significant correlation between gender and practitioners' knowledge about autism [66]. Male practitioners' attitudes toward autism significantly differed from those of female practitioners (*p* < 0.001). A previous study also revealed a significant correlation between gender and practitioners' attitudes regarding autism [67]. The average mean for males (16.3443) is comparatively higher than that of females (15.0811), indicating that heightened sensitivity to stimuli is among the traits associated with autism (Table 5).

According to the Pearson correlation test findings, the study found no significant correlation between GPs' age, years since graduation, and years of practice and their knowledge of autism. However, earlier research indicated that GPs' years of experience and age negatively correlated with their understanding of autism [65, 68, 69]. The study did find the following weakly significant relationships: GPs' age correlated with their views on autism (*r* = 0.271⁣^∗∗^, *p* < 0.001) and their years of practice also correlated with their views (*r* = 0.105⁣^∗^, *p*=0.046). In contrast, the study found no meaningful association between the years since graduation and GPs' perspectives on autism. Being familiar with and spending time with someone who has autism has a significant impact on attitudes toward autism. This seems to be a very effective strategy for enhancing these attitudes. Supporting this conclusion, a different study discovered that exposure to autistic individuals favorably influences attitudes toward autism [70, 71]. A cross-sectional survey in China showed that practitioners' attitudes toward autism did not significantly correlate with their age or years of practice [72]. In addition, this study found a small positive correlation (*r* = 0.004⁣^∗∗^, *p* < 0.001) between GPs' knowledge and their attitudes toward autism, which earlier research did not observe [64].

The KAP theory supports this conclusion by suggesting that gaining more knowledge can change attitudes, which then influence practice and behavior [73]. These studies' conclusions indicate that KAP-based training can advance and improve people's understanding, perspectives, and behaviors [74, 75]. We expect that possessing the appropriate information will be one of the traits that encourage positive behavioral changes, even if we accept that there are other elements that influence health practices.

### 4.1. Study Recommendations and Their Limitations

Our investigation has a number of limitations. This study's modest sample approach limits the generalizability of the results to a larger group. The cross-sectional study design prevents us from making causal inferences between the independent variables and the study outcomes. To improve the results and strengthen causal inferences and generalizations, researchers should conduct random sampling and cohort studies.

## 5. Conclusion

According to the survey, GPs' knowledge and attitudes toward autism were moderate. There were notable distinctions in the opinions of male and female practitioners (*p* < 0.001). The study found weakly significant connections between practitioners' years of practice and their attitudes (*r* = 0.105⁣^∗^, *p*=0.046) as well as between practitioners' age and their attitudes (*r* = 0.271⁣^∗∗^, *p* < 0.001). Furthermore, there was a weakly significant correlation (*r* = 0.004⁣^∗∗^, *p* < 0.001) between practitioners' attitudes regarding autism and their level of understanding. However, the study did not discover any meaningful relationships between the years of practice, age, or years since graduation and the practitioners' knowledge.

The results indicate a need for enhanced training and educational programs to improve GPs' knowledge and attitudes, which could positively impact patient care. Targeted CME courses and interactive workshops, along with gender-sensitive educational strategies, can address these gaps and promote more effective autism management.

Future research should focus on longitudinal studies to assess the long-term effects of training interventions, evaluate the impact of specific educational programs on knowledge and patient outcomes, and explore gender differences in attitudes toward autism. Expanding the study to include GPs from various regions can provide a broader perspective and guide the development of more comprehensive and effective educational initiatives. Addressing these areas will enhance GPs' ability to deliver high-quality care and improve outcomes for individuals with autism.

## Figures and Tables

**Table 1:** General characteristics of GPs surveyed (*n* = 360).

Variable		Subject (%)
Gender	Male	212 (58.9%)
	Female	148 (41.1%)

Age group (years)	< 31	184 (51.1%)
	31–40	120 (33.3%)
	≥ 40	56 (15.6%)
Graduation years group (years)	< 10	114 (31.7%)
	10–15	53 (14.7%)
	15–20	12 (3.3%)
	≥ 20	181 (50.3%)

Practice years group (years)	< 10	122 (33.9%)
	10–15	48 (13.3%)
	15–20	17 (4.7%)
	≥ 20	173 (48.1%)

**Table 2:** Knowledge and attitudes of the GPs towards autism (*n* = 360).

Questions	Number (%)				
	Strongly agree	Agree	Undecided	Disagree	Strongly disagree
Children with autism are often detached from their family and peers	93 (25.6%)	121 (33.6%)	42 (11.7%)	74 (20.6)	30 (8.3%)
Autism is a possible result of neglect by the parents	44 (12.2%)	57 (15.8%)	76 (21.1%)	131 (36.4%)	52 (14.4%)
Autism is a precursor for schizophrenia	56 (15.6%)	63 (17.5%)	112 (31.1%)	69 (19.2%)	60 (16.7%)
It is often difficult to distinguish between autism and schizophrenia	108 (30.0%)	45 (12.5%)	51 (14.2%)	19 (5.3%)	6 (1.7%)
Autism is more prevalent in higher socioeconomic classes	39 (10.8%)	69 (19.2%)	73 (20.3%)	101 (28.1%)	55 (15.3%)
Children with autism are not affectionate	39 (10.8%)	69 (19.2%)	73 (20.3%)	125 (34.7%)	54 (15.0)
Children can grow out of autism	31 (8.6%)	87 (24.2%)	71 (19.7%)	133 (36.9)	38 (10.6%)
Autism is more prevalent among higher educational classes	43 (11.9%)	96 (26.7)	48 (13.3%)	113 (31.4%)	60 (16.7%)
There is a stigma against autism in my community	65 (18.1%)	64 (17.8%)	70 (19.4%)	115 (31.9%)	46 (12.8%)
Autism is preventable	53 (14.7%)	79 (21.9%)	47 (13.1%)	103 (28.6%)	78 (21.7%)
Do you believe should autistic children receive special education	203 (56.4%)	100 (27.8%)	19 (5.3%)	22 (6.1%)	16 (4.4%)
Do you think autism holds a social stigma in this community	103 (28.6%)	95 (26.4%)	46 (12.8%)	67 (18.6%)	49 (13.6%)
Do you believe that diagnosing a child with autism will lead to discrimination against the child	73 (20.3%)	73 (20.3%)	14 (3.9%)	154 (42.8%)	46 (12.8%)
In general there is a negative opinion towards children diagnosed with autism	90 (25%)	95 (26.4%)	35 (9.7%)	79 (21.9%)	61 (16.9%)
Parents in Ethiopia tend to think their children are at risk of autism	12 (3.3%)	28 (7.8%)	36 (10.0%)	178 (49.4%)	106 (29.4%)

**Table 3:** Level classification for total scores of knowledge and attitude regarding autism.

Percentage of total scores	Total scores of knowledge	Total scores of attitude	Level
80–100	31–41	17–21	Good
60–79	29–31	15–17	Moderate/fair
<60	0–28	0–14	Poor

**Table 4:** Relationship between sociodemographic variables with total scores of knowledge and attitude as well as knowledge with attitude regarding autism (*n* = 360).

Variables	*r*	*p*
Age vs. knowledge	0.69	0.194
Age vs. attitude	0.271⁣^∗∗^	<0.001
Graduation year vs. knowledge	0.069	0.193
Graduation year vs. attitude	0.037	0.486
Practice year vs. knowledge	−0.053	0.312
Practice year vs. attitude	0.105⁣^∗^	0.046
Knowledge vs. attitude	0.004⁣^∗∗^	<0.001

*Note: *⁣^∗^ and ⁣^∗∗^ show the level of correlations.

**Table 5:** Comparison of total scores of knowledge and attitude regarding autism with gender using independent *t*-test (*n* = 360).

Total scores	Male mean (SD) (*n* = 212)	Female mean (SD) (*n* = 148)	*t*-statistics (df)	*p*
Knowledge	29.2877 (3.54086)	29.8986 (2.6283)	−1.783 (358)	0.075
Attitude	16.3443 (3.20387)	15.0811 (3.21669)	3.675 (358)	<0.001

## Data Availability

All the data generated during this study are included within the manuscript. The dataset of the study can be accessed without any restriction. The datasets generated and/or analyzed during the current study are not publicly available due to being part of an ongoing study, and we want to maintain exclusivity over the data until the study is complete or published, but they are available from the corresponding author upon reasonable request.

## References

[1] Zeidan J., Fombonne E., Scorah J. (2022). Global Prevalence of Autism: A Systematic Review Update. *Autism Research*.

[2] Bourgeron T. (2021). What Do We Know about Early Onset Neurodevelopmental Disorders. *Translational Neuroscience: Toward New Therapies*.

[3] Morris-Rosendahl D. J., Crocq M.-A. (2020). Neurodevelopmental Disorders—the History and Future of a Diagnostic Concept. *Dialogues in Clinical Neuroscience*.

[4] Tomé F. M. S. (2020). West Syndrome: From Etiology to Prognosis.

[5] Nwosu G. I. (2023). Developmental and Epileptic Encephalopathy Associated With Human GABRB3 Mutations Disrupt GABAA Receptor Configuration Resulting in Lennox-Gastaut Syndrome. *Gabrb3+/N328D Knock-In Mice*.

[6] Carter A. N. (2021). *Childhood Epilepsy and Internalizing Disorders: Reference Table of Screening Instruments*.

[7] Mohammed H. H. A. (2023). *Clinical Utility of Next-Generation Sequencing in Children With Cryptogenic Cerebral Palsy; A Cohort Study From a Tertiary Paediatric Neurology Clinic in the Western Cape Province of South Africa*.

[8] Soke G. N., Maenner M., Windham G. (2019). Association Between Breastfeeding Initiation and Duration and Autism Spectrum Disorder in Preschool Children Enrolled in the Study to Explore Early Development. *Autism Research*.

[9] Lin J., Costa M. A., Rezende V. L. (2024). Risk Factors and Clinical Profile of Autism Spectrum Disorder in Southern Brazil. *Journal of Psychiatric Research*.

[10] Adak B., Halder S. (2023). Prenatal, Perinatal and Postnatal Maternal Risk Factor for Autism Spectrum Disorder: Need to Understand the Genetic–Environment Intersect. *The Routledge Handbook of Inclusive Education for Teacher Educators: Routledge India*.

[11] Persico A. M., Merelli S. (2015). Environmental Factors and Autism Spectrum Disorder. *Autism Spectrum Disorders*.

[12] Mate A., Reyes-Goya C., Santana-Garrido Á, Sobrevia L., Vázquez C. M. (2021). Impact of Maternal Nutrition in Viral Infections During Pregnancy. *Biochimica et Biophysica Acta (BBA)-Molecular Basis of Disease*.

[13] Young M. F., Ramakrishnan U. (2020). Maternal Undernutrition Before and During Pregnancy and Offspring Health and Development. *Annals of Nutrition and Metabolism*.

[14] Adams J. B., Kirby J. K., Sorensen J. C., Pollard E. L., Audhya T. (2022). Evidence Based Recommendations for an Optimal Prenatal Supplement For women in the US: Vitamins and Related Nutrients. *Maternal Health, Neonatology and Perinatology*.

[15] Saroukhani S., Samms-Vaughan M., Lee M. (2020). Perinatal factors Associated With Autism Spectrum Disorder in Jamaican Children. *Journal of Autism and Developmental Disorders*.

[16] Bragg M., Chavarro J. E., Hamra G. B. (2022). Prenatal Diet as a Modifier of Environmental Risk Factors for Autism and Related Neurodevelopmental Outcomes. *Current environmental health reports*.

[17] Ashmawi N. S., Hammoda M. A. (2022). Early Prediction and Evaluation of Risk of Autism Spectrum Disorders. *Cureus*.

[18] Begum R., Mamin F. A. (2019). Impact of Autism Spectrum Disorder on Family. *Autism-Open Access*.

[19] Lyall K., Croen L., Daniels J. (2017). The Changing Epidemiology of Autism Spectrum Disorders. *Annual Review of Public Health*.

[20] Wu S., Wu F., Ding Y., Hou J., Bi J., Zhang Z. (2017). Advanced Parental Age and Autism Risk in Children: A Systematic Review and Meta‐Analysis. *Acta Psychiatrica Scandinavica*.

[21] Taufiq N., McKeithan G. K. (2024). Screening and Diagnostic Tools for Autism Spectrum Disorder Utilized in the Indian Subcontinent: A Scoping Review. *International Journal of Developmental Disabilities*.

[22] Shenouda J., Barrett E., Davidow A. L. (2023). Prevalence and Disparities in the Detection of Autism Without Intellectual Disability. *Pediatrics*.

[23] Kadhim R. A., Baban R. S., Al-Omrani A. A. A., Alhafidh K. H. (2024). Serum Levels of 25-OH Vitamin D3 and Vitamin D Receptor Among Iraqi Children With Autism Spectrum Disorder. *Advancements in Life Sciences*.

[24] Murphy M. S., Abdulaziz K. E., Lavigne É (2024). Association Between Prenatal Air Pollutant Exposure and Autism Spectrum Disorders in Young Children: A Matched Case-Control Study in Canada. *Environmental Research*.

[25] Schmidt R. J., Goodrich A. J., Delwiche L. (2024). Newborn Dried Blood Spot Folate in Relation to Maternal Self-Reported Folic Acid Intake, Autism Spectrum Disorder, and Developmental Delay. *Epidemiology*.

[26] Choueiri R., Garrison W. T., Tokatli V. (2023). Early Identification of Autism Spectrum Disorder (D): Strategies for Use in Local Communities. *Indian Journal of Pediatrics*.

[27] Talantseva O. I., Romanova R. S., Shurdova E. M. (2023). The Global Prevalence of Autism Spectrum Disorder: A Three-Level Meta-Analysis. *Frontiers in Psychiatry*.

[28] Marlow M., Servili C., Tomlinson M. (2019). A Review of Screening Tools for the Identification of Autism Spectrum Disorders and Developmental Delay in Infants and Young Children: Recommendations for Use in Low‐and Middle‐Income Countries. *Autism Research*.

[29] Nielsen T. C., Nassar N., Boulton K. A., Guastella A. J., Lain S. J. (2024). Estimating the Prevalence of Autism Spectrum Disorder in New South Wales, Australia: A Data Linkage Study of Three Routinely Collected Datasets. *Journal of Autism and Developmental Disorders*.

[30] Torres E. B., Twerski G., Varkey H. (2023). The Time is Ripe for The Renaissance of Autism Treatments: Evidence From Clinical Practitioners. *Frontiers in Integrative Neuroscience*.

[31] Alallawi B., Hastings R. P., Gray G. (2020). A Systematic Scoping Review of Social, Educational, and Psychological Research on Individuals With Autism Spectrum Disorder and Their Family Members in Arab Countries and Cultures. *Review Journal of Autism and Developmental Disorders*.

[32] Olusanya B. O., Davis A. C., Wertlieb D. (2018). Developmental Disabilities Among Children Younger Than 5 Years in 195 Countries and Territories, 1990–2016: A Systematic Analysis for the Global Burden of Disease Study 2016. *Lancet Global Health*.

[33] Alakhzami M., Huang A. (2023). Individuals With Autism Spectrum Disorders and Developmental Disorders in Oman: An Overview of Current Status. *Journal of Autism and Developmental Disorders*.

[34] Hai Y., Qu A., ul Ain Q., My Z., Lj W., LiJie W. (2023). A Comparison of the Epidemiological Factors and Burden of Autism Spectrum Disorders Worldwide and in China. *Austin Journal of Autism and Related Disabilities*.

[35] Svenaeus F. (2013). Diagnosing Mental Disorders and Saving the Normal: American Psychiatric Association. *Diagnostic and Statistical Manual of Mental Disorders*.

[36] Mishra R., Dargar S. K. (2023). Teaching Diversified Socio-Cultural Skills to Autism Spectrum Disorder (ASD) Students in Clinical Setting. *AIP Conference Proceedings*.

[37] Dwyer P., Williams Z. J., Lawson W. B., Rivera S. M. (2024). A Trans-Diagnostic Investigation of Attention, Hyper-Focus, and Monotropism in Autism, Attention Dysregulation Hyperactivity Development, and the General Population. *Neurodiversity*.

[38] Alsaedi R. H., Carrington S., Watters J. J. (2023). Caregivers’ assessment of the Sensory Processing Patterns Exhibited by Children With Autism in the Gulf Region. *Journal of Autism and Developmental Disorders*.

[39] Baweja R., Waschbusch D. A., Mayes S. D. (2023). Physical Aggression Toward Others and Self: Correlates in Autism, Attention-Deficit/Hyperactivity Disorder, and Population-Based Child Samples. *JAACAP Open*.

[40] Fatma Z. S. (2021). Assessment of Knowledge and Practice About Childhood Autism Among Health Care Workers at Kenyatta National Hospital: UON.

[41] Joon P., Kumar A., Parle M. (2021). What is Autism?. *Pharmacological Reports*.

[42] Eta E. V. (2022). Healthcare Issues in Children With Developmental Disabilities (Autism). *Pediatrics and Neonatal Nursing—Open Journal*.

[43] Maynard D. W., Turowetz J. (2022). *Autistic Intelligence: Interaction, Individuality, and the Challenges of Diagnosis*.

[44] Auberry K. (2022). Educating Behavior Clinicians in a Community Behavior Care Center for Children With Autism Spectrum Disorder: Medication Administration a Pilot Study in the United States. *Journal of Intellectual Disabilities*.

[45] Shrestha C., Shrestha A., Joshi J., Karki S., Acharya S., Joshi S. (2021). Does Teaching Medical Ethics Ensure Good Knowledge, Attitude, and Reported Practice? An Ethical Vignette-Based Cross-Sectional Survey Among Doctors in a Tertiary Teaching Hospital in Nepal. *BMC Medical Ethics*.

[46] Jayalath M., Nanayakkara R., Jayasundara K., Abeysekara C., Abeynayake A. (2021). Knowledge, Attitudes and Practices Towards Medical Ethics Among Specialist Medical Officers of Selected Allopathic Healthcare Institutions in Sri Lanka: Conocimiento, Actitudes y prácticas hacia la ética médica entre los Funcionarios médicos Especialistas de Instituciones Sanitarias Alopáticas Seleccionadas en Sri Lanka. *South Florida Journal of Health*.

[47] Thangavelu P. D., Janakiraman B., Pawar R. (2024). Understanding, Being, and Doing of Bioethics; a State-Level Cross-Sectional Study of Knowledge, Attitude, and Practice Among Healthcare Professionals. *BMC Medical Ethics*.

[48] Shetty A. K., Vaswani R. (2022). Knowledge, attitude and Practice of Healthcare Ethics Among Final Year Medical and Nursing Students at a College in South India. *Biomedicine*.

[49] Carbone A., Dell’Aquila A. (2023). The Diagnosis of Pervasive Developmental Disorder Not Otherwise Specified: A Systematic Literature Review. *Children*.

[50] O’Sharkey K., Mitra S., Paik S., Chow T., Cockburn M., Ritz B. (2024). Trends in the Prevalence of Autism Spectrum Disorder in California: Disparities by Sociodemographic Factors and Region Between 1990–2018. *Journal of Autism and Developmental Disorders*.

[51] O’Reilly M., Lester J. N., Kiyimba N. (2020). Autism in the Twentieth Century: An Evolution of a Controversial Condition. *Healthy Minds in the Twentieth Century: In and beyond the Asylum*.

[52] Krieger I., Grossman-Giron A., Comaneshter D. (2021). The Co-Occurrence of Autistic Spectrum Disorder and Schizophrenia: A Nationwide Population-Based Study. *Journal of Psychiatric Research*.

[53] Whitehead P. (2022). Kurt Goldstein’s Critique of Leo Kanner: Understanding Autism as an Impairment of the Abstract Attitude. *Early Child Development and Care*.

[54] Lutz A. S. (2024). *Chasing the Intact Mind: How the Severely Autistic and Intellectually Disabled Were Excluded From the Debates That Affect Them Most*.

[55] Schelly D. (2021). Parental Action and Referral Patterns in Spatial Clusters of Childhood Autism Spectrum Disorders. *Encyclopedia of Autism Spectrum Disorders*.

[56] Clauser P., Ding Y., Chen E. C., Cho S.-J., Wang C., Hwang J. (2021). Parenting Styles, Parenting Stress, and Behavioral Outcomes in Children With Autism. *School Psychology International*.

[57] Nantanda R., Kayingo G., Jones R., van Gemert F., Kirenga B. J. (2020). Training Needs for Ugandan Primary Care Health Workers in Management of Respiratory Diseases: A Cross Sectional Survey. *BMC Health Services Research*.

[58] Mulupi S., Ayakaka I., Tolhurst R. (2022). What Are the Barriers to the Diagnosis and Management of Chronic Respiratory Disease in Sub-Saharan Africa? A Qualitative Study With Healthcare Workers, National and Regional Policy Stakeholders in Five Countries. *BMJ Open*.

[59] Rahbar M. H., Dobrescu I., Gillani S. (2021). Factors Associated With Knowledge, Attitude, and Practices of Physicians Related to Autism Spectrum Disorder in Romania.

[60] Unigwe S., Buckley C., Crane L., Kenny L., Remington A., Pellicano E. (2017). GPs’ Confidence in Caring for Their Patients on the Autism Spectrum: An Online Self-Report Study. *British Journal of General Practice*.

[61] Bellando J., Fussell J. J., Lopez M. (2016). Autism Speaks Toolkits: Resources for Busy Physicians. *Clinical Pediatrics*.

[62] Hyman S. L., Levy S. E., Myers S. M. (2020). Identification, Evaluation, and Management of Children With Autism Spectrum Disorder. *Pediatrics*.

[63] Minhas A., Vajaratkar V., Divan G. (2015). Parents’ Perspectives on Care of Children With Autistic Spectrum Disorder in South Asia–Views From Pakistan and India. *International Review of Psychiatry*.

[64] van t Hof M., van Berckelaer-Onnes I., Deen M. (2020). Novel Insights Into Autism Knowledge and Stigmatizing Attitudes Toward Mental Illness in Dutch Youth and Family Center Physicians. *Community Mental Health Journal*.

[65] Garg P., Lillystone D., Dossetor D., Kefford C., Chong S. (2014). An Exploratory Survey for Understanding Perceptions, Knowledge and Educational Needs of General Practitioners (GSs) Regarding Autistic Disorders in New South Wales (NSW), Australia. *Journal of Clinical and Diagnostic Research: Journal of Clinical and Diagnostic Research*.

[66] Kuzminski R., Netto J., Wilson J., Falkmer T., Chamberlain A., Falkmer M. (2019). Linking Knowledge and Attitudes: Determining Neurotypical Knowledge About and Attitudes Towards Autism. *PLoS One*.

[67] Corden K., Brewer R., Cage E. (2022). A Systematic Review of Healthcare Professionals’ Knowledge, Self-Efficacy and Attitudes Towards Working With Autistic People. *Review Journal of Autism and Developmental Disorders*.

[68] Rahbar M. H., Ibrahim K., Assassi P. (2011). Knowledge and Attitude of General Practitioners Regarding Autism in Karachi, Pakistan. *Journal of Autism and Developmental Disorders*.

[69] Hend M. S. (2017). Assessment of Family Physicians’ Knowledge of Childhood Autism. *Family Medicine and Community Health*.

[70] Dickter C. L., Burk J. A. (2021). The Effects of Contact and Labeling on Attitudes Towards Individuals With Autism. *Journal of Autism and Developmental Disorders*.

[71] Kim S. Y., Song D. Y., Bottema‐Beutel K., Gillespie‐Lynch K., Cage E. (2023). A Systematic Review and Meta‐Analysis of Associations Between Primarily Non‐Autistic People’s Characteristics and Attitudes Toward Autistic People. *Autism Research*.

[72] Mao S., Fan X., Ma Y., Chen Y., Lv J., Yang R. (2022). Knowledge and Beliefs About Autism Spectrum Disorders Among Physicians: A Cross-Sectional Survey From China. *BMJ Paediatrics Open*.

[73] Verplanken B., Orbell S. (2022). Attitudes, Habits, and Behavior Change. *Annual Review of Psychology*.

[74] Wang J., Chen L., Yu M., He J. (2020). Impact of Knowledge, Attitude, and Practice (KAP)-Based Rehabilitation Education on the KAP of Patients With Intervertebral Disc Herniation. *Annals of Palliative Medicine*.

[75] Yang J., Yang J., Guo D., Zhao Q., Chen Y. (2022). Outcome of Nursing Based on Health Belief United With Knowledge, Belief, and Practice Mode on Gastroscopy of Patients With Gastric Cancer. *Computational and Mathematical Methods in Medicine*.

